# Heterogeneous Optical Fiber Sensor System for Temperature and Turbidity Assessment in Wide Range

**DOI:** 10.3390/bios12111041

**Published:** 2022-11-18

**Authors:** Arnaldo Leal-Junior, Guilherme Lopes, Leandro C. Macedo, Welton Duque, Anselmo Frizera, Carlos Marques

**Affiliations:** 1 Graduate Program of Electrical Engineering of Federal, University of Espírito Santo, Vitória 29075-910, Brazil; 2 Mechanical Engineering Department of Federal, University of Espírito Santo, Vitória 29075-910, Brazil; 3 Physics Department & I3N, University of Aveiro, 3810-193 Aveiro, Portugal

**Keywords:** intensity variation sensors, turbidity, temperature, fiber Bragg gratings, surface plasmon resonance

## Abstract

This paper presents the development of an optical fiber sensor system for multiparametric assessment of temperature and turbidity in liquid samples. The sensors are based on the combination between fiber Bragg gratings (FBGs), intensity variation and surface plasmon resonance (SPR) sensors. In this case, the intensity variation sensors are capable of detecting turbidity with a resolution of about 0.5 NTU in a limited range between 0.02 NTU and 100 NTU. As the turbidity increases, a saturation trend in the sensor is observed. In contrast, the SPR-based sensor is capable of detecting refractive index (RI) variation. However, RI measurements in the turbidity calibrated samples indicate a significant variation on the RI only when the turbidity is higher than 100 NTU. Thus, the SPR-based sensor is used as a complementary approach for the dynamic range increase of the turbidity assessment, where a linearity and sensitivity of 98.6% and 313.5 nm/RIU, respectively, are obtained. Finally, the FBG sensor is used in the temperature assessment, an assessment which is not only used for water quality assessment, but also in temperature cross-sensitivity mitigation of the SPR sensor. Furthermore, this approach also leads to the possibility of indirect assessment of turbidity through the differences in the heat transfer rates due to the turbidity increase.

## 1. Introduction

Optical fiber sensors are electrically passive and have electromagnetic immunity [1]. As a result, they are superior to electrical-based sensors (piezoelectric, piezoresistive, and capacitive sensors) for procedures that require high magnetic fields (such as magnetic resonance imaging) [2], electric motors assessment [3] and applications in classified areas [4]. Additionally, some sterilization methods rely on high heat, pressure, and humidity, which can damage the electronic circuits of sensors; optical sensors, however, are more resistant to these effects [5]. They are also compact, lightweight, chemically stable, and capable of multiplexing, making them suitable for a variety of applications [6].

In comparison to conventional electrical transducers, optical fiber sensors offer many well-known and desirable characteristics for label-free methods [7]. There are many advantages to this technology, including its size, immunity to electromagnetic interference, cost, light path control, remote sensor deployment, high transmission rates, ability to hold multiple sensors on a single fiber, and the use of biocompatible materials that are intrinsically safe and inert, thereby reducing their environmental impact [8]. These advantages have led to the use of optical fiber sensors in a variety of fields including medicine [9], environmental monitoring [10], and antibody detection [11]. Optical fiber sensors are selected for such applications due to their operational safety in aqueous environments, as well as the ease with which they can be introduced into the tanks, avoiding the need to collect samples for testing on external instruments [12]. Moreover, sensing devices may be either hand-held probes or a set of remote-controlled devices attached to an optical fiber cable.

The development of optical fiber sensors has been characterized by a variety of approaches, including intensity variation-based sensors [13], interferometry [14], fiber Bragg gratings (FBG) [15], tilted FBG (TFBG) [16], surface plasmon resonance (SPR)-based sensors [17], long period gratings (LPG) [18], and distributed sensors using optical fiber nonlinear effects [6].

Every ecosystem relies on water for daily survival, as it is a vital component of life on earth. In spite of the fact that water is an abundant resource on earth, pollution and waste lead to a substantial reduction in the amount of water available for human consumption, causing a water shortage in some regions [19]. A variety of sources contribute to water pollution, including chemical disposal of pharmaceuticals, industrial processes, agriculture, and household waste [20]. In light of the importance of water and the increasing pollution in rivers, oceans, and lakes, the promotion and assessment of water quality have become key priorities in environmental policy [19]. Moreover, water pollution also impacts aquatic species, which are exposed to environmental pollution, resulting in an increase in mortality [20]. In addition to polluting aquatic species, the toxins in fish and other aquatic species can also be transmitted to other aquatic and non-aquatic species, including humans, via the food chain [21].

Water turbidity is defined as the degree to which particles in the water disrupt the passage of light [22]. As a result, turbidity is a measure of water clarity since it reflects the extent to which suspended particles in the water impair the ability to see clearly. These particles can be considered sediment, which includes a wide variety of matter including soil particles, algae, plankton, and microorganisms [22]. Water color can be changed by these particles, which are extremely small [23].

Due to the particles absorbing sunlight, high turbidity increases the water temperature. Water at higher temperatures contains less oxygen, resulting in hypoxic conditions [24]. Due to these higher temperatures, fish use more oxygen due to increased metabolic rates, thereby further limiting the oxygen supply. Considering the environmental impact, light is scattered by suspended particles, preventing it from reaching plants and algae, thus further reducing oxygen levels. In general, different optical fiber sensing technologies have been proposed for the assessment of temperature [25,26] and turbidity [27]. For turbidity assessment, most of the technologies are based on the intensity variation of the transmitted optical signal in the medium [28]. However, complementary methods based on the thermal and refractive index assessment for turbidity measurement have not been thoroughly explored using optical fiber sensing approaches.

Aiming at the necessity and significance of simultaneous monitoring of turbidity and temperature, this paper presents the development of a novel heterogeneous optical fiber probe for simultaneous assessment of turbidity and temperature using the wavelength and reflected optical power data. The sensor can effectively measure the turbidity and temperature in a large range through the combination of different optical fiber sensing techniques, namely intensity variation, FBGs and SPR.

## 2. Materials and Methods

The sensor system is based on a heterogeneous optical fiber sensor structure to obtain a sensor system that can effectively measure temperature and turbidity for a wide range of samples, from 0 to 4000 NTU. The sensor structure is based on three different operation principles, namely intensity variation, FBG and SPR, where each operation principle presents superior performance in a range of turbidity. In addition, there is the possibility of sensor fusion between the data for the achievement of a sensor with higher accuracy, dynamic range and resolution.

**Figure 1:**
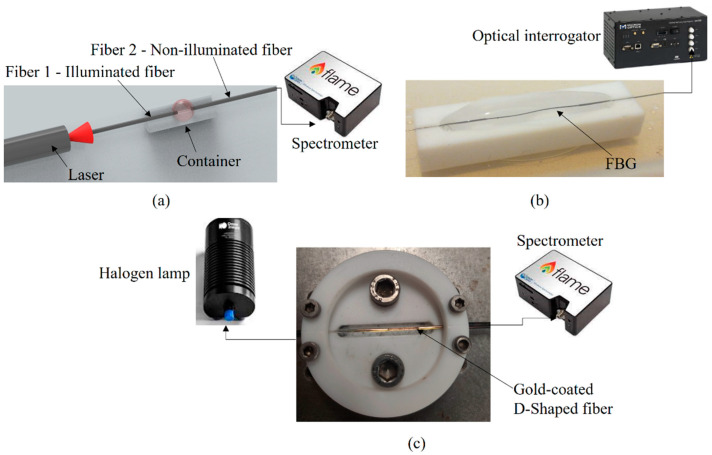
Experimental setup for (**a**) intensity, (**b**) FBG, and (**c**) SPR.

For the first approach on turbidity estimation, an intensity variation sensor is proposed. Such sensors are based on a well-known principle of optical power attenuation between two fibers separated by a medium [29]. The turbidity of the liquid samples between the optical fibers can be estimated from the transmitted optical power variation for each turbidity sample. In this case, one of the fibers is connected to the light source, whereas the other is connected to a photodetector (or spectrometer), see Figure 1a. Thus, the turbidity increase between both optical fibers leads to a reduction of the transmitted optical power between illuminated and non-illuminated fibers.

For the FBG-based sensor, the uniform grating was inscribed in photosensitive single mode silica fiber. The uniform FBGs were produced using a pulsed Q-switched Nd:YAG laser system (LOTIS TII LS-2137U), emitting the fourth harmonic (266 nm) [30], with an emission power lamp energy of 26 J and measured pulse energy of 120 J with a repetition rate of 1 Hz. The laser beam profile was circular with the diameter around 8 mm and divergence less than 1.0 mrad. An effective focal length of 320 mm was used to focus the laser beam onto the fiber core. On the fiber surface, the beam produced a spot size of 8 mm wide and 30 µm high. The phase mask employed was 10 mm long with a pitch of 1064 nm, designed for 266 nm irradiation, resulting in an FBG with Bragg wavelength of 1544 nm.

As FBGs are well-known for their temperature sensitivity, the temperature sensor was based on the direct application of the optical fiber (with inscribed FBGs) in the liquid sample. Thus, the temperature was directly evaluated through the heat transfer from the liquid to the FBG [31]. Furthermore, since the turbidity directly affects the liquid thermal properties, it is possible to estimate the turbidity from the thermal dynamics in the liquid. For this reason, the FBG sensor was used not only as a temperature sensor, but also for the turbidity estimation through the thermal dynamics of the fluid obtained from the transient analysis of the FBG response (as presented in [32]). Figure 1b shows the experimental setup for the evaluation of the FBG responses at different fluid turbidity conditions.

The D-shape in the optical fiber was performed to expose the optical fiber core prior to the gold deposition for the SPR signal. To clarify the D-shape in the optical fiber, an in-house method was performed. With the in-house home-made method, a segment of a polymer optical fiber (POF), made of polymethyl methacrylate (PMMA) material with a total diameter (core and cladding) of 1 mm, was cut to a length of approximately 30 cm and clamped on two sides, each on opposite sides of the fiber, so that it remained stretched and steady throughout the entire polishing procedure. Using a plier and a blade, a portion of the black plastic jacket covering the PMMA was removed and placed on the V-groove to be polished. This section of removed plastic must have a length greater than the polishing blade, which has a length of one centimeter, so usually five centimeters of plastic are removed.

In the final step before polishing began, the POF was connected to a light source on one end and a power meter on the other end, so that power loss could be monitored during the polishing process. There is a custom V-groove in which the POF was placed. This groove has a depth of approximately 700 µm, leaving approximately 300 µm of the PMMA uncovered to be removed by the polishing machine.

The polishing machine was held by a claw, which was attached to a 3D platform, the V-groove, on which the POF was placed. During the polishing process, both the claw and the V-groove were moved in three directions to ensure that the polishing machine was parallel to the fiber, the D-Shape was centered, and the sides of the fiber were not polished.

Between each step of the SPR sensor fabrication, i.e., the D-shape and the gold deposition, the fiber samples were cleaned with deionized water. A thin Au film, 50 nm thick, was sputtered onto the D-shaped POF samples. For the coating process, first, the fiber was cleaned with isopropanol and then placed in the sputtering chamber (SEM coating Unit E5000 mounted with a sputter target composed of 99.99% Au). An Au layer was deposited on one side of the fiber (in the D-shaped area). By controlling the deposition time, the thickness of the Au layer was estimated. Furthermore, previous tests on the nanolayer thickness for this target were conducted using a scanning electron microscope, where the film thickness was assessed for different deposition times. Subsequently, to enhance the adhesion of the Au on the surface of the POFs, the Au-coated POFs were annealed for two hours at 50 °C.

The sensor characterizations were performed as a function of the turbidity (for the intensity variation sensor), temperature (for the FBG sensor) and refractive index (for the SPR sensor). In the case of the intensity variation sensor, the transmitted optical power decreases as the turbidity increases. However, the sensor signal can reach a saturation point in which the changes in the turbidity do not lead to further variations in the transmitted optical power. For this reason, the intensity variation sensor was tested on calibrated samples with turbidities of 0.02 NTU, 20 NTU, 100 NTU and 800 NTU with the sensor positioned inside a Teflon container for mechanical protection of the sensor probe. As shown in Figure 1a, the controlled samples were positioned inside the container and a laser centered at 662 nm was connected to fiber 1 (illuminated fiber), whereas fiber 2 (non-illuminated fiber) was connected to a spectrometer with a detection range from 180 nm to 890 nm (FLAME-T-UV–vis, manufactured by Ocean Optics, Orlando, FL, USA).

For the temperature characterization of the FBG, the sensor was positioned inside a climatic chamber with controlled temperature. The test was performed in a range of 25 °C to 45 °C in steps of 5 °C. Thereafter the thermal dynamic tests were performed in 3 different samples, 0.02 NTU, 20 NTU and 60 NTU under temperature variation conditions. In this case, 100 mL of each sample was subjected to a temperature step variation from 25 °C to 50 °C and their transient temperature variations were measured by the FBG sensor as presented in Figure 1b. All the data from the FBG were acquired by the optical interrogator sm125 (Micron Optics, Atlanta, GA, USA).

For the RI characterization, the D-shaped, gold-coated POF was positioned in a container similar to that of the intensity variation-based sensor in which the samples with different RI were positioned. Figure 1c shows the experimental setup for this test. The characterization of the SPR sensor includes the RI variation from 1.3398 to 1.3681 and was arrived at by filling the container with different liquid samples. In the tests, one end of the fiber was connected to a halogen lamp and the other end to the spectrometer.

Thus, all three principles can detect the turbidity from direct or indirect measurements (related to RI and thermal dynamics differences). For this reason, the principles can be combined for a simultaneous measurement of turbidity and temperature in a larger range, where the intensity variation sensor can be used for smaller ranges of turbidity variation (from 0.02 NTU to 800 NTU), whereas the SPR sensor can be used in higher ranges (from 800 NTU to 4000 NTU) in which there is a higher variation of the RI. Concurrently, the FBG sensor not only provides a real time and continuous monitoring of the temperature, but is also capable of detecting the turbidity from the transient response of the temperature variation in the sample, since higher turbidities lead to faster heat transfer dynamics.

## 3. Results and Discussions

**Figure 2:**
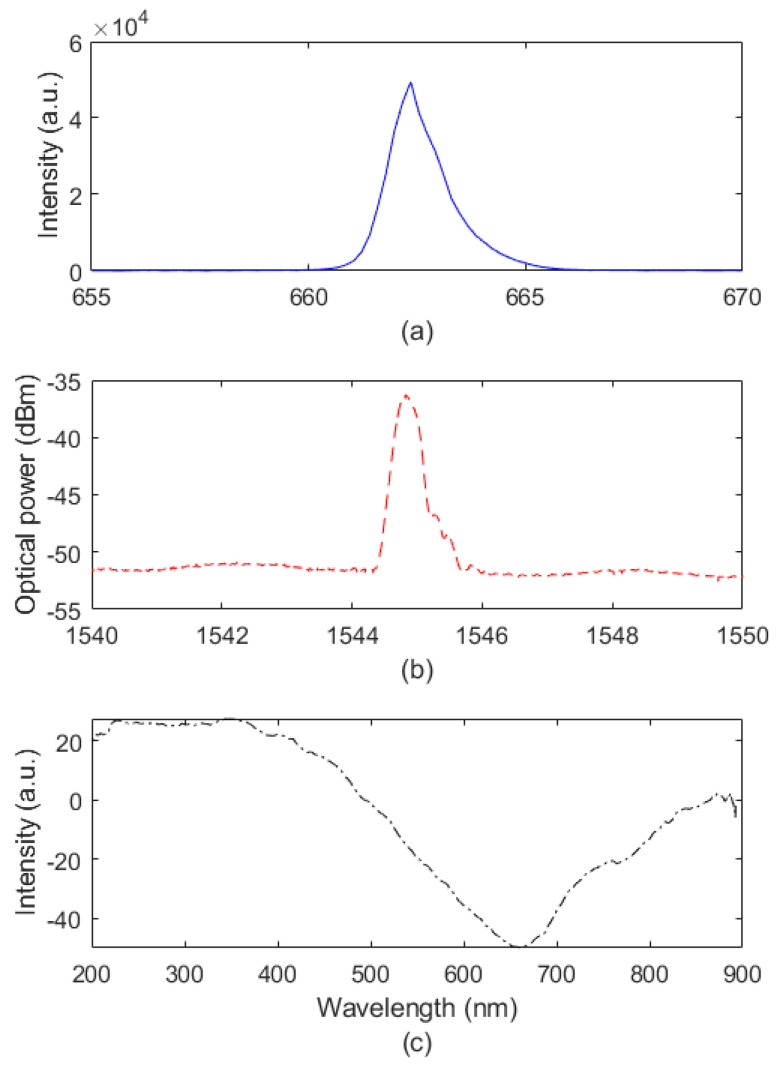
Transmitted/reflected spectrum for (**a**) intensity, (**b**) FBG, and (**c**) SPR.

Figure 2 presents the spectral responses of all proposed sensors, where it is possible to observe in Figure 2a that the transmitted spectrum of the intensity variation sensor is only the narrow peak centered on the laser center wavelength. Figure 2b shows the reflected spectrum of the FBG used for the thermal assessment of the liquids, whereas Figure 2c shows the transmitted spectrum of the SPR sensor, which presented an SPR signature at around 680 nm. As shown in Figure 2, the sensors operate at different wavelength regions and are individually characterized using the materials discussed in Section 2. It is also worth mentioning that the FBG sensor was inscribed in silica optical fiber, whereas the SPR and intensity variation sensors were used in PMMA POFs.

**Figure 3:**
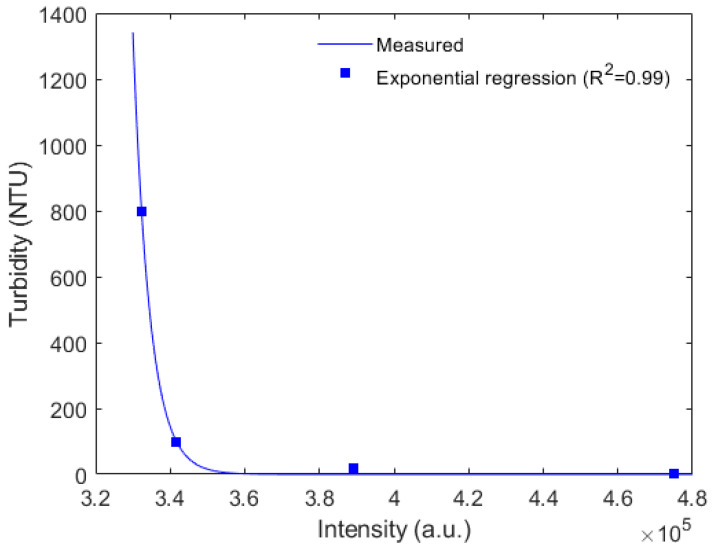
Turbidity as a function of optical power variation for the intensity variation-based sensor.

Following the characterizations discussed in Section 2, Figure 3 presents the optical power variation as a function of the turbidity for samples in the range of 0.02 NTU to 800 NTU, the optical power is estimated from the integral of the transmitted spectrum for each turbidity condition. The results in Figure 3 indicate a saturation trend on the sensor response when the turbidity reaches 800 NTU, which leads to a limitation on the sensor operation in terms of turbidity range. The intensity variation sensor presented a high determination coefficient (R^2^) with an exponential regression (R^2^ of 0.99).

**Figure 4:**
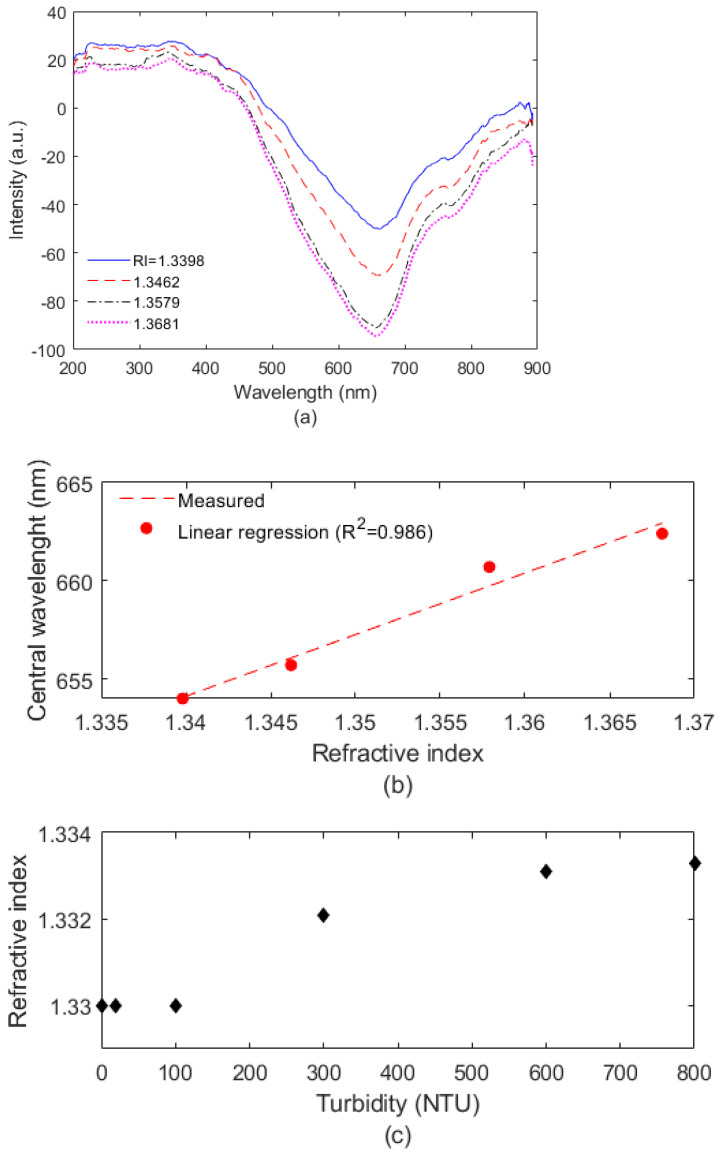
(**a**) Transmitted spectra, (**b**) linear regression., (**c**) RI for turbidity samples.

The optical fiber sensor behavior on the turbidity assessment indicates the necessity of complimentary approaches to increase the dynamic range of the sensor, which can also impact its resolution and accuracy. As the first complementary approach, the SPR sensor is analyzed as a function of the RI. In this case, the liquid samples with different RI are placed in the container and the transmitted spectra are analyzed for each case, as shown in Figure 4a. The tests were performed at constant temperature conditions of 23 °C. The results indicate a variation in both intensity and wavelength of the optical signal, Figure 4b shows a regression of the optical power and wavelength of the SPR signal as a function of the RI, where it is possible to observe a linear trend in the response with an R2 of around 0.98. The results indicate the possibility of using the SPR-based sensor for the RI assessment, which can be used for the estimation of the samples turbidity. In this case, Figure 4c shows the refractive index measured by a benchtop refractometer for the samples of different turbidity. These results show that the RI of the turbidity samples start to change at around 100 NTU, since there is no RI variation in the turbidities below 100 NTU. The RI is around 1.330 for the turbidities of 0.02 NTU, 20 NTU, and 100 NTU. Then, the RI increases in the samples of 300 NTU, 600 NTU and 800 NTU, where the results indicate an RI of 1.3333 for 800 NTU. Thus, the use of the SPR sensor as a complementary approach for the turbidity assessment increases the dynamic range of the whole sensing system, since the SPR sensor can be used for the turbidity assessment with NTU higher than 100.

**Figure 5:**
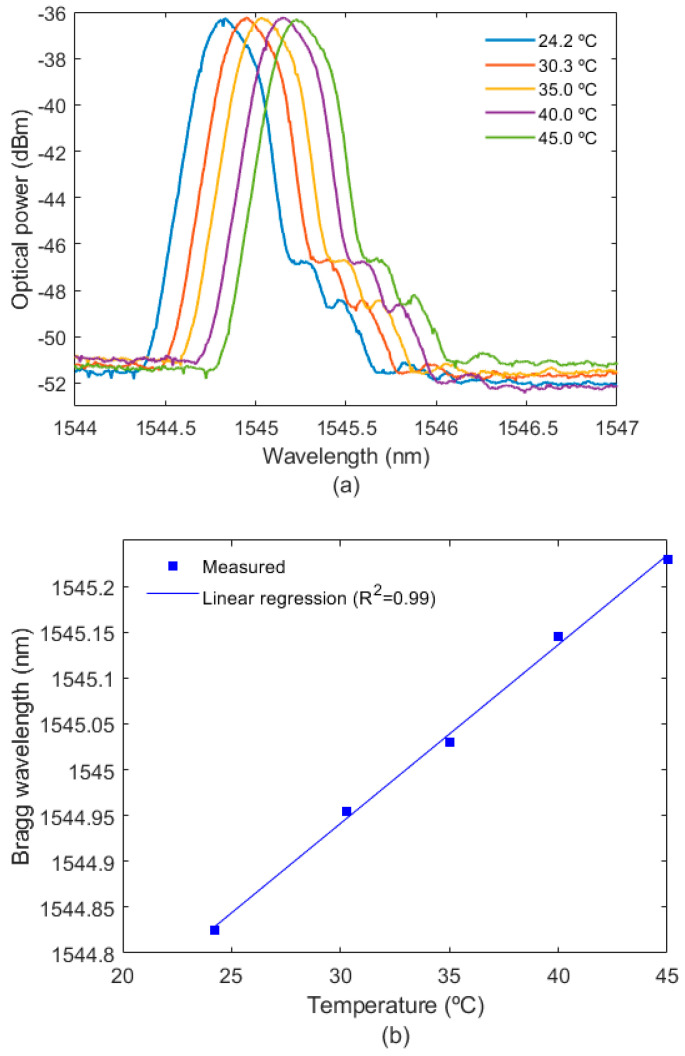
(**a**) FBG spectra as a function of the temperature, (**b**) Bragg wavelength shift for different temperatures.

The SPR sensor also has temperature sensitivity, which leads to the necessity of temperature monitoring for the mitigation of the temperature cross-sensitivity. In order to evaluate the temperature of the liquid in which the sensors are immersed, the FBG temperature sensor is characterized as a function of the temperature variation. The results of the temperature tests and their influence on the reflected spectrum of the sensing device are presented in Figure 5a, in which it is possible to observe that there are only wavelength shifts on the reflected optical spectrum. Figure 5b shows the wavelength shift as a function of the temperature for the characterization tests, where there is a well-known high linearity (R^2^ = 0.999) and a sensitivity of around 10 pm/°C, as commonly obtained in FBG-based temperature sensors.

**Figure 6:**
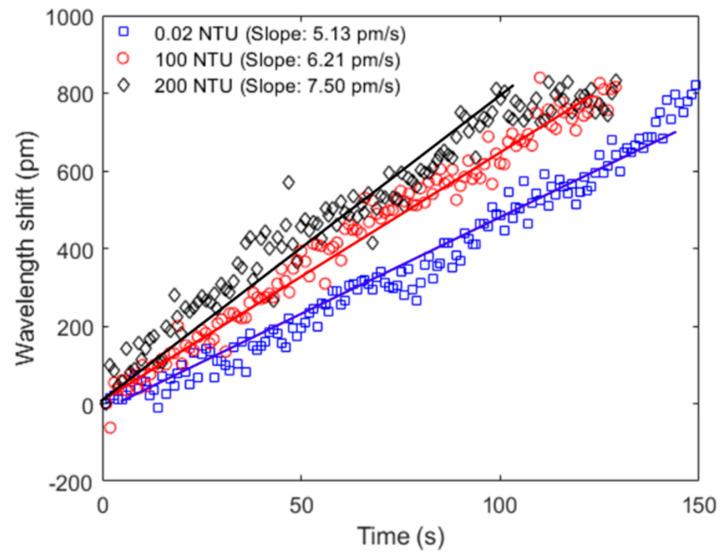
Transient response of the temperature for three different samples. The temperature is estimated using the FBG sensors.

The continuous temperature assessment through the FBG sensor also leads to the possibility of estimating the temperature dynamics of the liquid samples. As liquids with higher turbidity have larger temperature variations due to the particles absorbing sunlight, the transient thermal responses of the samples can indicate their turbidity. In order to verify this, three different samples (turbidities of 0.02 NTU, 100 NTU and 200 NTU) were subjected to temperature variations, whereas the temperature response of each sample was acquired with the FBG temperature sensor. It is important to mention that we used three different samples, since the turbidity variation of a single sample leads to the need to stir the sample for a time to obtain a homogeneous solution, which can affect the transient thermal behavior needed for the turbidity estimation from temperature response and will lead to results different from those expected in a practical application in which there is no forced convection in the sample. Figure 6 presents the results of the transient thermal response of each sample, where the temperature increases from 25 °C to around 40 °C. The slope of the curve related to transient analysis as a function of the time indicates the rate of heat transfer to the samples. For this reason, higher slopes indicate higher rates of heat transfer, which is related to the sample turbidity, as shown in Figure 6. Therefore, it is possible to estimate the turbidity of each sample from the heat transfer rate between the samples. As it is an indirect estimation of the turbidity, the transient thermal analysis can be used as another complementary approach for turbidity assessment in conjunction with the intensity variation-based sensors and SPR sensing approaches.

The results indicate different (and complementary) possibilities of measuring turbidity in a large range. However, it is also possible to integrate such sensors, especially the intensity variation sensor and the FBG, for the simultaneous assessment of temperature and turbidity using a single optical fiber probe.

It is also worth mentioning that the SPR sensor can be integrated in the probe. In this case, the FBG should be inscribed in the visible wavelength region and the transmitted spectrum also needs to be acquired. Moreover, a D-shape and gold coating in the fiber is needed, where the sensor has the same operating principle as discussed in Section 2, i.e., the SPR signature is analyzed as a function of the RI (related to the sample turbidity). Thus, this approach can result in the integration of all proposed sensors in a single heterogeneous optical fiber probe.

## 4. Conclusions

This paper presented the development, characterization and analysis of a heterogeneous optical fiber sensing structure for simultaneous assessment of turbidity and temperature. The sensor system is composed of three optical fiber sensing approaches: (i) an intensity variation sensor for the direct monitoring of turbidity through the transmitted optical power variation; (ii) an FBG temperature sensor for continuous and real time monitoring of thermal parameters, which can also be used for indirect estimation of the turbidity; and (iii) an SPR-based sensor for turbidity assessment through RI variations. The developed sensors presented complementary behavior, where the FBG temperature sensor can be used for temperature cross-sensitivity mitigation in SPR sensors as well as assessment of fluid thermal properties. The SPR sensor can operate in a larger range of turbidities (from values higher than 100) through the refractive index variations of the samples, whereas the intensity variation sensor presented a high resolution (about 0.5 NTU) in the turbidity assessment in the range of 0.02 NTU to 100 NTU. Therefore, the proposed sensors can be combined for turbidity assessment with higher accuracy and dynamic range, where signal processing techniques can be employed in the data fusion. In addition, the sensors can be integrated into a single probe for compact and multiplexed analysis. Future works will include the use of the proposed device in real application scenarios of environmental monitoring.

## Data Availability

Not applicable.
